# Satisfaction with Telemedicine for Cancer Pain Management: A Model of Care and Cross-Sectional Patient Satisfaction Study

**DOI:** 10.3390/curroncol29080439

**Published:** 2022-08-04

**Authors:** Marco Cascella, Sergio Coluccia, Mariacinzia Grizzuti, Maria Cristina Romano, Gennaro Esposito, Anna Crispo, Arturo Cuomo

**Affiliations:** 1 Department of Anesthesia and Critical Care, Istituto Nazionale Tumori—IRCCS, Fondazione Pascale, 80100 Naples, Italy; 2 Epidemiology and Biostatistics Unit, Istituto Nazionale Tumori—IRCCS, Fondazione Pascale, 80131 Naples, Italy

**Keywords:** telemedicine, cancer pain, patient satisfaction, workflow, protocol

## Abstract

Background: Since cancer pain requires complex modalities of care, the proper strategy for addressing its telemedicine-based management should be better defined. This study aimed to trace a pathway for a progressive implementation of the telemedicine process for the treatment of pain in the setting of cancer patients. Methods: The features of the model were investigated to dissect the dropout from the telemedicine pathway. A cross-sectional patient satisfaction study was conducted. The degree of satisfaction was evaluated through a developed 22-item questionnaire (Likert scale 0–7). Results: A total of 375 video consultations for 164 patients (mean age 62.9 years) were performed through remote consultations for cancer pain management between March 2021 and February 2022. After the exclusion of 72 patients, 92 (56.1%) were included in the analysis. The dropout ratio was 8.7%. The number of visits and pharmacological therapies for neuropathic pain correlated with the risk for readmission (*p* < 0.05). Overall, the satisfaction was very high (mean > 5.5 for all items). Conclusion: Feedback from patients reflected high satisfaction rates with the care provided. A methodological approach based on the degree of satisfaction combined with the analysis of the pathways can help to implement the quality of a service provided through telemedicine. While not without limitations, our hybrid protocol can be useful for addressing cancer pain through a patient-centered approach.

## 1. Introduction

In recent years, the implementation of telemedicine strategies has been observed in multiple fields of medicine for diagnosis, treatment, disease prevention, research, and education. Obviously, the COVID-19 pandemic stimulated the development and application of different modalities for the remote delivery of healthcare services. Remote monitoring, real-time, and store-and-forward processes have been rapidly implemented worldwide [1]. In Italy, several laws have been enacted for integrating telemedicine services into the national health organization, and the regulatory system has the perspective of including these strategies within the essential levels of care guaranteed by the public health system [2].

Several attempts have been conducted on the use of telemedicine for pain management [3,4], also in cancer patients [5]. These studies focused on the outcomes of clinical efficacy and health policy arguments. For example, it was shown that, compared with traditional in-person consultations, healthcare services at a distance improved access to care and facilitated the continuity of care [6]. Furthermore, telemedicine methods can allow a better distribution of resources and contain costs without negatively affecting performance quality [7].

Nevertheless, within the chronic pain chapter, cancer pain represents a subset that should usually be addressed through complex modalities of care. Cancer patients suffering from chronic pain must be managed through multidisciplinary, extended, and skilled strategies. This complexity may often pose challenges for healthcare organizations. Moreover, in these patients, the issues of pain management add to the numerous clinical needs these patients commonly require [8].

Consequently, it is necessary to design a detailed pathway for better encompassing the telemedicine strategy in the whole therapeutic process. The proper use of technology, privacy issues, and modalities for remote doctor–patient interaction, as well as the ability to ensure access for an in-person visit, are examples of the key elements to be investigated.

In the lack of large-scale experiences and precise recommendations from scientific societies, defining and improving a telemedicine process for cancer pain can take advantage of patients’ feedback. Combined with information obtained from the developed pathways for patients’ care, these inputs can be used to identify and correct workflow and technical problems.

This study aimed to evaluate the adherence to the telemedicine pathway and to obtain patients’ feedback. The adherence to the telemedicine model was investigated through a set of variables. Moreover, the patient satisfaction analysis was performed by using a developed questionnaire. The collected data can be used for a progressive implementation of the telemedicine process for the treatment of cancer pain and its effective integration into routine care. In this study, the proposed model of care refers to a synchronous real-time communication telemedicine process (video consultation) combined with a store-and-forward system for providing the secure electronic transmission of patient data.

## 2. Methods

### 2.1. Study Population

This is a cross-sectional patient satisfaction study conducted at the Istituto Nazionale Tumori, Fondazione Pascale, Italy, in accordance with the Declaration of Helsinki and approved by the local Medical Ethics Committee (protocol code 41/20 Oss; date of approval, 26 November 2020). All patients provided written informed consent.

Adult cancer patients with painful conditions who entered the cancer pain clinic were offered the opportunity to have one or more subsequent remote consultations. For patients with poor health status or those unfamiliar with technology, the involvement of a skilled caregiver was required. The inclusion criteria for remote pain management are shown in Table 1.

### 2.2. Questionnaire

The adopted questionnaire was assembled by combining items from a validated questionnaire (i.e., Telehealth Usability Questionnaire (TUQ)) with further questions (*n* = 5). The TUQ collects questions from existing telehealth instruments with those from computer usability tools. It is a 17-item comprehensive tool that covers all usability elements including usefulness, effectiveness, reliability, and satisfaction. The questionnaire can be used with different telehealth modalities, such as conventional video systems, computer-based modalities, and the latest mobile telehealth approaches [9].

Five other questions were developed to address privacy issues, features of medical-patient interaction, the patient’s involvement in the therapeutic decision-making process, the efficacy of telemedicine, and the patient’s capability to express pain characteristics through telemedicine. These additional questions were designed following Peterson’s brief, relevant, unambiguous, specific, and objective (BRUSO) strategy [10].

The questions of the composed questionnaire were answered using a 7-point Likert scale (i.e., from “strongly disagree” = 1, to “strongly agree” =7) (Table 2).

### 2.3. Model of Care

#### 2.3.1. Information Technology (IT) Infrastructure

To guarantee the security of the acquired data and privacy, an ad hoc platform was structured. This tool was developed starting from the oncology network of the Campania Region (southern Italy). It is commonly used for the management of cancer patients throughout the territory. The in-house IT staff trained in the secure management of patient data has integrated this platform with the regional health information system designed to support the entire Campania Regional Health Service (Sinfonia) [11]. The booking system provides access to the Cancer Institute’s portal. An operator collects the reservation and enters it in the calendar. A linked connection is established between healthcare providers and patients/caregivers. The patient can send all the necessary clinical documentation, which is uploaded on the platform. Importantly, access to patient data is strictly limited to the operator, as secure login to the platform is required.

#### 2.3.2. Operational Phases: The Hybrid Model of Care

The IT infrastructure allows all the regulatory processes, including reservation, generation, and sending of the link (via email) for connection, and data collection (e.g., imaging, laboratory, tests, clinical data, etc.). It also guarantees data security.

During the first in-person visit, the preparation for telemedicine is performed. This phase includes legal and regulatory issues (consent), data collection, and training. Detailed information to the patient (and caregiver) on the possibility of performing visits remotely is provided. Furthermore, this preliminary step is essential to acquire information on the patient’s access to the equipment (e.g., laptop) and ability to manage IT supports. Finally, a training phase is carried out. This preliminary step is also fundamental for establishing a relationship with the patient, and for diagnostic or therapeutic purposes. Tools for pain and quality of life evaluation can be provided. The subsequent real-time video consultation is planned according to the clinical need.

The care needs of patients who access telemedicine are the same as those of patients who are managed in the Pain Clinic. These needs include titration of treatment, pharmacological re-evaluation, management of side effects, and other clinical problems. The management is carried out according to the protocols used in common clinical practice. During the remote follow-up, clinicians interact with the patient and caregiver, evaluate the clinical condition, and assess the provided data. Furthermore, other specialists can participate in the remote visit by invitation, to carry out a multiprofessional consultation. According to clinical needs, the process involves integration with oncologists, radiotherapists, physiatrists, psychologists, and other professionals. The platform also allows professionals from outside the Cancer Institute to be included among the participants. Consequently, if required, clinical assessments can also be carried out in collaboration with primary care specialists and personnel of the territorial palliative care team.

After the visit, medical reports are emailed to the patient. Other scheduled remote controls are programmed but in-person visits can be required to carry out minimally invasive procedures, for diagnosis, and other motivations. In the case of acute clinical scenarios (e.g., probable bone lesion), rapid access to the hospital is provided. A dynamic (hybrid) process allows for rapid in-person reassessment, for example, for patients who require interventional techniques. Moreover, in addition to scheduled visits, the patient can request additional visits both in telemedicine and in person.

For the study aims, the questionnaires were administered via telephone by a trained operator (M.G., lawyer and expert in policy and scientific research processes) after the first visit (within a maximum of 10 days and never after a second visit).

**Figure 1:**
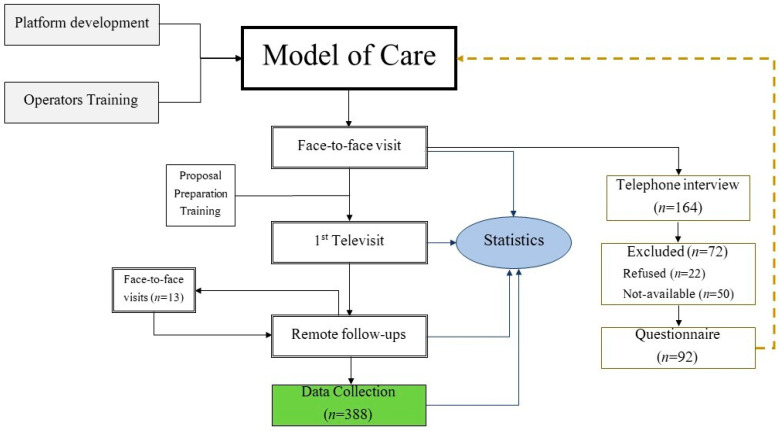
Model of care and the study flowchart. The model of care is designed through the structuring of the platform and the training of the operators. The first visit is carried out in person. It allows for collecting consent, clinical evaluation, and patient training. After the remote visit, follow-ups are scheduled. They can be delivered through telemedicine, but readmission to the clinic is provided. The satisfaction questionnaire is administered via telephone after the first remote visit. The answers and the descriptive analysis serve as feedback to improve the whole process. The collected data on 388 remote visits can be used for clinical investigations and the development of ad hoc predictive models.

A flow diagram of the model of care and the study process is illustrated in Figure 1.

### 2.4. Statistical Analysis

Data were analyzed using the R software, version 4.1.3 (R Foundation for Statistical Computing, Vienna, Austria). The toolkit included the MICE Suite for imputation of missing data. The univariate analysis to assess potential associations with dropout was performed through the Wilcoxon rank-sum test for numeric variables, the chi-square test for categorical variables, and Fisher’s exact test for binary variables. A multivariate logistic model was adopted to evaluate the association between the potential risk factors and dropout; the model was adjusted for confounders. For the latter aim, a stepwise technique was used.

## 3. Results

A total of 375 video consultations for 164 patients (81 females) with a mean age of 62.9 (±11.6) years old were performed through remote consultations for cancer pain management between March 2021 and February 2022. Of the 164 patients, 72 were excluded (refused *n* = 22; not available for the interview *n* = 50); finally, 92 (56.1%) patients were eligible for the descriptive analysis and the patient satisfaction study (Figure 1).

Most patients (*n* = 52) had more than one visit. The average number of visits was 2.4 per patient. Eight patients (8.7%) interrupted the telemedicine pathway (dropouts) and were re-evaluated in person. The motivations were the need for an invasive procedure (*n* = 4) or a clinical assessment (*n* = 4). No patient requested an in-person visit.

The univariate analysis was performed to investigate the dropout phenomenon. It demonstrated that the number of visits correlated with the risk for readmission (Table 3). Since dropout can occur regardless of the number of visits (even after the first visit), we considered all patients potentially at risk of abandoning the remote visit pathway.

The multivariate analysis showed a significantly increased risk of dropout in patients treated with neuropathic pain medications (*p* = 0.043). Although not significant, the male gender was associated with an increased risk for readmission (*p* = 0.068) (Table 4).

**Figure 2:**
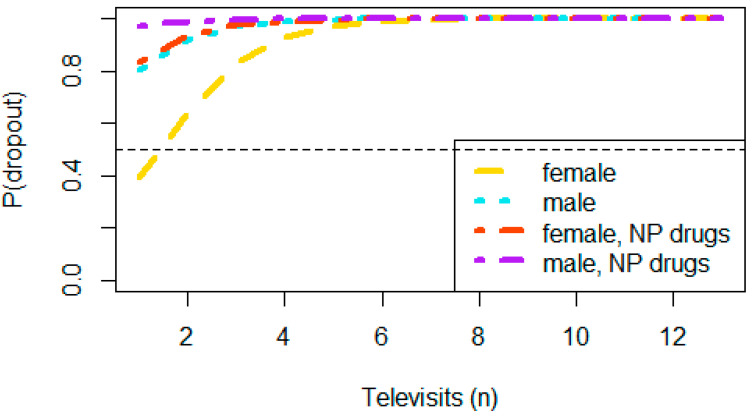
Probability of in-person visit. The probability of dropout (*p*) increased as the number of visits increased and especially in male patients who were treated with drugs for neuropathic pain (e.g., anticonvulsants and antidepressants). Abbreviations: NP, neuropathic pain.

The correlation between gender, pharmacological therapies neuropathic pain, number of remote visits, and risk for hospital or ambulatory readmission is provided in Figure 2.

**Figure 3:**
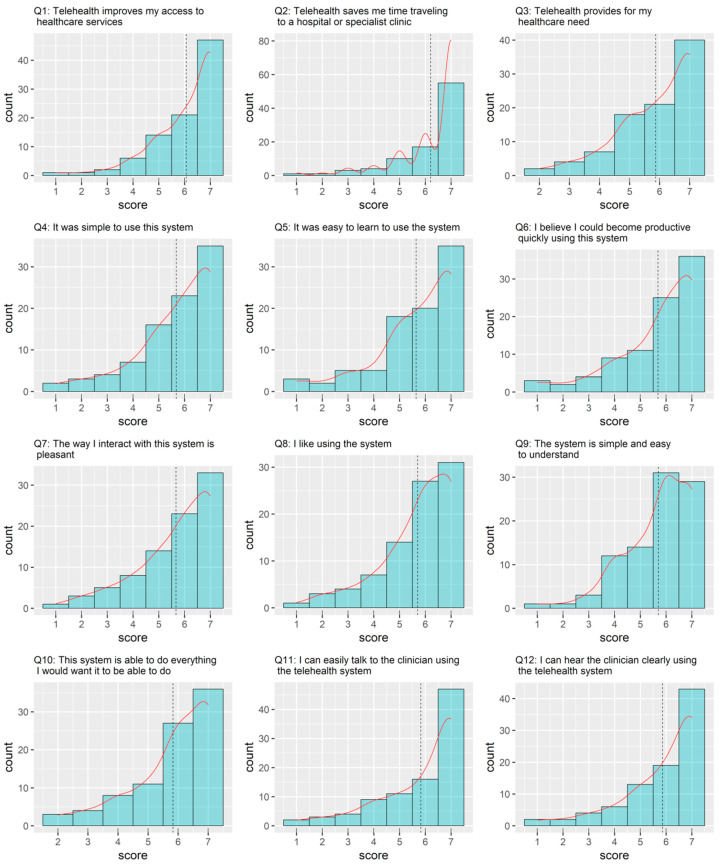
Patient satisfaction questionnaire (items 1–12). The questions were answered using a 7-point Likert scale (i.e., from “strongly disagree” = 1, to “strongly agree” = 7). The dashed lines indicate the mean values.

**Figure 4:**
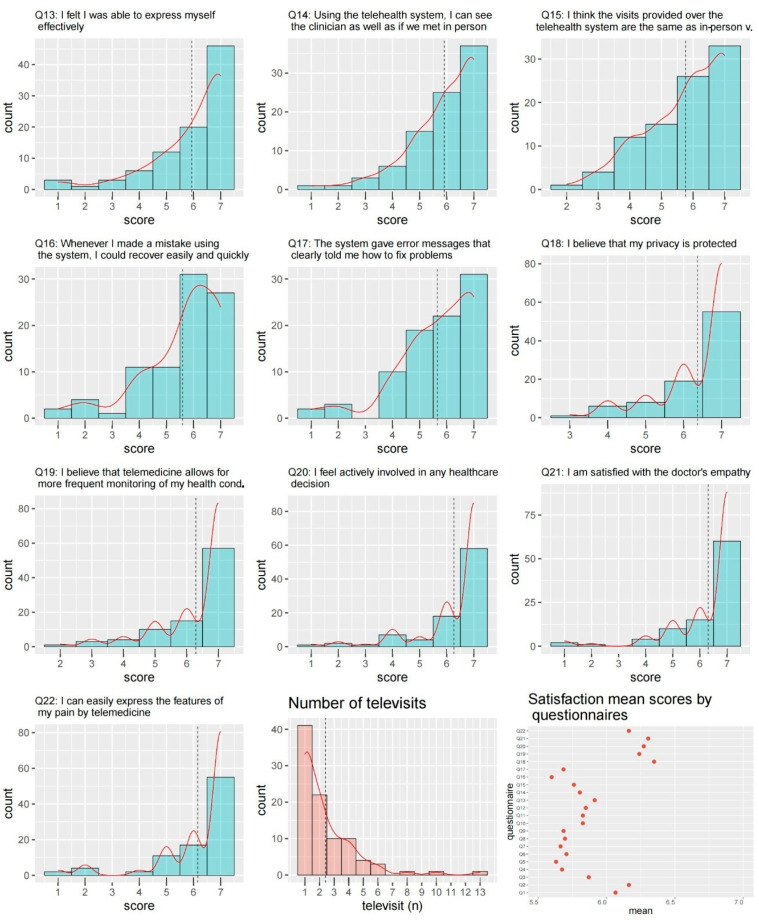
Patient satisfaction questionnaire (items 13–22). The questions were answered using a 7-point Likert scale (i.e., from “strongly disagree” = 1, to “strongly agree” = 7). The dashed lines indicate the mean values. The figure also reports the number of video consultations for a patient and the mean values for each item.

The results of the questionnaire on patient satisfaction are shown in Figure 3 and Figure 4. Overall, the satisfaction was very high, with a mean of more than 5.5 for all items (Figure 4).

## 4. Discussion

A remote system is a considerable possibility to promote access to care and continuing assistance. Nevertheless, in the setting of cancer patients, it is essential to structure a pathway that allows adapting the functionality of the process to the patient’s needs. The aim is to personalize treatments and increase therapeutic adherence. A recent literature analysis demonstrated that there is a gap in current knowledge on personalized approaches to address facilitators and barriers for remote consultations in cancer patients [12]. To fill this gap, it is necessary to collect experiences and establish accurate pathways. Subsequently, the pathway can be further refined by integrating telemonitoring and eHealth therapy approaches useful, for example, for managing pain and other symptoms in cancer survivors [13].

Although telemedicine for the treatment of cancer pain appears to be a resource with extensive potential applications, its implementation presents several obstacles. Research must necessarily fill various gaps and clarify how to structure a model that offers guarantees of effectiveness. The first problem to be faced concerns the IT infrastructure. Several telemedicine platforms are available [14]. The essential requirements of each platform are an operating system for the management of the whole service, devices (e.g., laptops), and an integrated software system (software modules) for sending documents, and imaging data. The IT system must provide the agile management of all phases of the process (reservations, contacts, links for connection, and data collection) and above all guarantee data security and privacy. Technical support and training must be provided to the staff to facilitate the use of technology. In our experience, we benefited from a platform used for the management of the COVID-19 pandemic. It was adapted to our needs, maintaining the original properties for data security. Despite these advantages, the platform needs appropriate corrections. For example, a solid integration is necessary to allow the management of patients who are subsequently assisted in the context of home palliative care. Another technical aspect to be implemented concerns the possibility of simplifying the communication processes through the development of ad hoc applications, for instance, for pharmacological management or side effects reporting.

The proposed model of care provides a first in-person visit. The need to carry out the first visit “preferably” in person is foreseen by the recent Italian legislation on telemedicine [3]. Nevertheless, in the absence of well-defined recommendations, we referred to our previous Delphi investigation [15] and to a recent nationwide survey on cancer pain management [16]. Notably, almost all the experts on the Delphi panel affirmed that, in such an important problem, preliminary face-to-face access should be required for clinical and regulatory purposes [15]. The same suggestion was collected in the subsequent survey [16]. This first face-to-face-visit step is also important for training, as patients and/or caregivers must be capable to use the telemedicine system. This approach was successfully adopted to design care models in populations with various medical issues [17,18].

The descriptive analysis showed interesting data. Cancer pain is an ongoing challenge for clinicians and requires detailed clinical (and diagnostic) reassessment. As regards therapy, multimodal drug strategies must be frequently combined with non-drug approaches. In these terms, dropout is not necessarily to be intended as a failure of telemedicine. In our study, this parameter was used to characterize the phenomenon of readmission and evaluate patients and clinical contexts that require greater attention. For example, this approach can be used to intercept patients who require a follow-up pathway with shorter time frames. Importantly, no patient requested an in-person visit.

Not surprisingly, the use of neuropathic pain medications was associated with a significant risk of readmission. This type of pain is difficult to treat with drugs, and most often, non-drug interventions are needed. These approaches for neuropathic pain are integrated into the treatment process. At the same time, it is intuitive that the number of visits was a major risk factor for dropout. For each visit, this risk increased by 30%. Patients who require a greater commitment of visits are defined as complex patients, as the painful symptoms are associated with drug side effects (e.g., opioid constipation) and other clinical problems. Consequently, they may require a more detailed clinical assessment. On the other hand, the performance status was not a risk factor. Furthermore, although not significant, males tended to leave the telemedicine process over 6 times more than women. Finally, the variable age had an opposite trend, and for each additional year, the risk decreased by 5%. Additional investigations are warranted to obtain confirmation and better explain these results. Other studies are also mandatory to establish the framework for a comprehensive telemedicine examination in this clinical setting.

Feedback from patients reflected high satisfaction rates with the care delivered. Probably, the possibility of establishing a relationship of trust with patients (and possibly caregivers) during the first visit facilitated the task of the operators. The positive response to questions regarding the platform and the functioning of the system was also important. It underlines the efforts in the preliminary phase of telemedicine planning. Other studies investigated the operators’ satisfaction with the platform and the developed model of care. In our case, as there were only three operators, a study to evaluate their feedback was not feasible [19].

The advantages of a hybrid model that combines face-to-face visits and remote follow-ups are undoubted. The degree of satisfaction, the containment of costs, and the possibility of carrying out an approach tailored to patients’ needs seem to be the main advantages. On the other hand, for operators, this strategy requires the important commitment of human and material resources. In our experience, we found a progressive increase in the demand for telemedicine consultations, even after the end of the COVID-19 pandemic. Paradoxically, outpatient visits did not undergo numerical decreases. In other words, through telemedicine services, new care needs were intercepted. This interesting finding needs to be supported by further studies. If confirmed, a reassessment of the resources needed to manage the increase in demand is required.

Finally, the disadvantages of the lack of standardized application strategies emerge. Telematic consultations were highly appreciated at the start of the COVID-19 crisis, but now that the pandemic is over, the pros and cons should be clearly regulated [20]. Careful guidelines and protocols to ensure modalities of telemedicine and the proper balance between in-person visits and remote consultations are warranted [6,21].

### Limitations

This study has several limitations. Information that is missing in the paper primarily concerns the level of pain of the participants in the study in the pre-, and post-telemedicine stages, as well as other data such as trends in the pain medications and clinical outcomes. Since in the literature, there is a serious lack of research on patient satisfaction with telemedicine in cancer pain, the study was focused on the degree of satisfaction with the process provided. In this view, we followed the research strategy widely used in other settings [22].

In the evaluation of patient satisfaction, the possible variability of the degree of satisfaction during the care process can represent a serious bias. For this reason, we chose a rigid criterion, and the interviews were conducted within 10 days from the first remote visit and never after a second remote consultation. We followed the same approach adopted in other research studies on telemedicine. For example, Pakanati et al. [23] carried out the survey within the second week of implementing telemedicine.

Some items of the questionnaire are conditioned by the operator. For example, question 21 “*I am satisfied with the doctor–patient communication*” refers to the clinician’s empathy. However, the telemedicine visits were carried out by three experienced physicians (G.E., M.C. and A.C.) with more than ten years of service as cancer pain therapists.

Only the first part of the questionnaire is validated (TUQ). Nonetheless, the further five questions were drawn through an analytical process (BRUSO). This was an attempt to cover all the critical aspects derived from a remote healthcare provider–patient interaction, as indicated in the literature [24,25]. Furthermore, it was underlined that the TUQ items can be modified to better address problems concerning both participants (operators and patients) and the telemedicine system [10].

The lack of operator feedback is another major limitation. The telemedicine process was developed collectively by the staff of the Pain Clinic in collaboration with the legal department and the IT staff. In these terms, we solved the various problems as a team through various meetings and multiprofessional discussions.

Additionally, we underline that, due to the small sample size, this study requires further investigations to confirm its preliminary results. Finally, no implementation or evaluation frameworks were used in the study design. In addition to the degree of satisfaction, the evaluation of the process should consider different parameters aimed at defining the appropriate corrective measures. To remedy this gap, a study based on artificial intelligence is ongoing. The satisfaction analysis combined with predictive investigations will allow us to design a more accurate telemedicine model.

## 5. Conclusions

The proposed model of care seems to be a valid approach for addressing pain problems in cancer patients. A methodological approach based on the degree of satisfaction combined with the analysis of the pathways can help to implement the quality of a service provided through telemedicine. The result is a flexible process that includes a scheduled follow-up program and possible readmissions to the hospital or outpatient clinic. While not without limitations, our protocol can be useful for addressing cancer pain through a patient-centered approach. Based on these findings, further research is needed to ensure the rapid implementation of telemedicine in well-designed cancer pain management pathways.

## Figures and Tables

**Table 1:** Inclusion criteria for remote pain management.

Cancer patients aged ≥ 18 years
2. No restriction for cancer disease
3. Availability of the necessary equipment for the video consultation (smartphone or laptop with webcam)
4. Ability to use the platform
5. Availability of a caregiver for patients with poor health status or those unfamiliar with the technology
6. Consent provided

**Table 2:** The adopted 22-item questionnaire.

1	Telehealth improves my access to healthcare services.
2	Telehealth saves me time traveling to a hospital or specialist clinic.
3	Telehealth provides for my healthcare need.
4	It was simple to use this system.
5	It was easy to learn to use the system.
6	I believe I could become productive quickly using this system.
7	The way I interact with this system is pleasant.
8	I like using the system.
9	The system is simple and easy to understand.
10	This system is able to do everything I would want it to be able to do.
11	I can easily talk to the clinician using the telehealth system.
12	I can hear the clinician clearly using the telehealth system.
13	I felt I was able to express myself effectively.
14	Using the telehealth system, I can see the clinician as well as if we met in person.
15	I think the visits provided over the telehealth system are the same as in-person visits.
16	Whenever I made a mistake using the system, I could recover easily and quickly.
17	The system gave error messages that clearly told me how to fix problems.
18	I believe that my privacy is protected.
19	I believe that telemedicine allows for more frequent monitoring of my health conditions.
20	I feel actively involved in any healthcare decision.
21	I am satisfied with the doctor–patient communication.
22	I can easily express the features of my pain through telemedicine.

Note: The questions were answered using a 7-point Likert scale (i.e., from “strongly disagree” = 1, to “strongly agree” = 7). Questions 1–17 from the Telehealth Usability Questionnaire (TUQ) [9]. The other questions were designed by following the BRUSCO strategy [10].

**Table 3:** Univariate analysis.

		Dropout		
Variable	Overall, *N* = 92 ^†^	No, *N* = 84 ^†^	Yes, *N* = 8 ^†^	*p*-Value ^‡^
**Age**				0.2
Mean (SD)	64 (12)	64 (12)	57 (16)	
Median (IQR)	66 (55, 73)	66 (57, 73)	52 (44, 72)	
**Gender**				0.15
F	48 (100%)	46 (96%)	2 (4.2%)	
M	44 (100%)	38 (86%)	6 (14%)	
**Neoplasm**				0.6
Other cancers	40 (100%)	36 (90%)	4 (10%)	
Colon	26 (100%)	23 (88%)	3 (12%)	
Breast	15 (100%)	15 (100%)	0 (0%)	
Lung	11 (100%)	10 (91%)	1 (9.1%)	
**Metastatic status**				0.15
No	48 (100%)	46 (96%)	2 (4.2%)	
Yes	44 (100%)	38 (86%)	6 (14%)	
**ECOG-PS**				>0.9
ECOG < 3	49 (100%)	45 (92%)	4 (8.2%)	
ECOG = 3	43 (100%)	39 (91%)	4 (9.3%)	
**Televisits** (*n*)				0.032
*n*	92	84	8	
Mean (SD)	2.4 (2)	2.2 (1.9)	4.1 (2.9)	
**MED**				>0.9
≤60	40 (100%)	37 (92%)	3 (7.5%)	
>60	52 (100%)	47 (90%)	5 (9.6%)	
**IV-Morphine**				0.4
No	87 (100%)	80 (92%)	7 (8.0%)	
Yes	5 (100%)	4 (80%)	1 (20%)	
**NP Drugs**				0.074
No	52 (57%)	50 (60%)	2 (25%)	
Yes	40 (43%)	34 (40%)	6 (75%)	

^†^ *n* (%); ^‡^ Wilcoxon rank-sum test; Fisher’s exact test. Abbreviations: ECOG-PS, Eastern Cooperative Oncology Group Performance Status; MED, morphine equivalent dose; IV-Morphine, intravenous morphine; NP, neuropathic pain.

**Table 4:** Multivariate analysis.

Characteristic	OR	95% CI	*p*-Value
Age	0.94	0.87, 1.01	0.10
Gender			
F	-	-	
M	6.36	0.99, 60.9	0.068
Metastatic status			
No	-	-	
Yes	4.92	0.81, 51.1	0.12
NP Drugs			
No	-	-	
Yes	7.68	1.25, 75.5	0.043
Televisits (*n*)	1.33	0.98, 1.84	0.062

Multivariate logistic regression: formula: logit (*p* (Dropout = T|X)) ~ Age + Gender + Metastatic status + NP Drugs + televisits. Abbreviations: OR, odds ratio; CI, confidence interval; NP, neuropathic pain.

## Data Availability

The data presented in this study are available on request from the corresponding author.

## References

[1] Hincapié M.A., Gallego J.C., Gempeler A., Piñeros J.A., Nasner D., Escobar M.F. (2020). Implementation and Usefulness of Telemedicine During the COVID-19 Pandemic: A Scoping Review. J. Prim. Care Community Health.

[2] Jalilian L., Wu I., Ing J., Dong X., Sadik J., Pan G., Hitson H., Thomas E., Grogan T., Simkovic M. (2022). Evaluation of Telemedicine Use for Anesthesiology Pain Division: Retrospective, Observational Case Series Study. JMIR Perioper Med..

[3] Italian Ministry of Health Organizational Guidelines on Home Care. https://www.gazzettaufficiale.it/eli/gu/2022/05/24/120/SG/html.

[4] Harnik M.A., Blättler L., Limacher A., Reisig F., Grosse Holtforth M., Streitberger K. (2021). Telemedicine for chronic pain treatment during the COVID-19 pandemic: Do pain intensity and anxiousness correlate with patient acceptance?. Pain Pract..

[5] Rocque G.B., Halilova K.I., Varley A., Williams C.P., Taylor R.A., Masom D.G., Wright W.J., Partridge E.E., Kvale E.A. (2017). Feasibility of a Telehealth Educational Program on Self-Management of Pain and Fatigue in Adult Cancer Patients. J. Pain Symptom Manag..

[6] Cascella M., Marinangeli F., Vittori A., Scala C., Piccinini M., Braga A., Miceli L., Vellucci R. (2021). Open Issues and Practical Suggestions for Telemedicine in Chronic Pain. Int. J. Environ. Res. Public Health.

[7] Baughman D., Ptasinski A., Baughman K., Buckwalter N., Jabbarpour Y., Waheed A. (2022). Comparable Quality Performance of Acute Low-Back Pain Care in Telemedicine and Office-Based Cohorts. Telemed. J. e-Health.

[8] Chua I.S., Zachariah F., Dale W., Feliciano J., Hanson L., Blackhall L., Quest T., Curseen K., Grey C., Rhodes R. (2019). Early integrated telehealth versus in-person palliative care for patients with advanced lung cancer: A study protocol. J. Palliat. Med..

[9] Parmanto B., Lewis A.N., Graham K.M., Bertolet M.H. (2016). Development of the Telehealth Usability Questionnaire (TUQ). Int. J. Telerehabil..

[10] Peterson R. Constructing Effective Questionnaires. http://methods.sagepub.com/book/constructing-effective-questionnaires.

[11] Campania Region. Piattaforma Sinfonia. https://sinfonia.regione.campania.it.

[12] Pang N.Q., Lau J., Fong S.Y., Wong C.Y., Tan K.K. (2022). Telemedicine Acceptance Among Older Adult Patients with Cancer: Scoping Review. J. Med. Internet Res..

[13] Knegtmans M.F., Wauben L.S.G.L., Wagemans M.F.M., Oldenmenger W.H. (2020). Home Telemonitoring Improved Pain Registration in Patients with Cancer. Pain Pract..

[14] Singh A.D. (2022). Telemedicine Workflow and Platform Options: What Would Work Well for Your Practice?. Clin. Liver Dis..

[15] Cascella M., Miceli L., Cutugno F., Di Lorenzo G., Morabito A., Oriente A., Massazza G., Magni A., Marinangeli F., Cuomo A. (2021). A Delphi Consensus Approach for the Management of Chronic Pain during and after the COVID-19 Era. Int. J. Environ. Res. Public Health.

[16] Cascella M., Vittori A., Petrucci E., Marinangeli F., Giarratano A., Cacciagrano C., Tizi E.S., Miceli L., Natoli S., Cuomo A. (2022). Strengths and Weaknesses of Cancer Pain Management in Italy: Findings from a Nationwide SIAARTI Survey. Healthcare.

[17] Alexander V.M., Schelble A.P., Omurtag K.R. (2021). Traits of patients seen via telemedicine versus in person for new-patient visits in a fertility practice. F S Rep..

[18] Odden J.L., Khanna C.L., Choo C.M., Zhao B., Shah S.M., Stalboerger G.M., Bennett J.R., Schornack M.M. (2020). Telemedicine in long-term care of glaucoma patients. J. Telemed. Telecare.

[19] Vosburg R.W., Robinson K.A., Gao C., Kim J.J. (2022). Patient and Provider Satisfaction with Telemedicine in a Comprehensive Weight Management Program. Telemed. J. e-Health.

[20] Crispo A., Montagnese C., Perri F., Grimaldi M., Bimonte S., Augustin L.S., Amore A., Celentano E., Di Napoli M., Cascella M. (2020). COVID-19 Emergency and Post-Emergency in Italian Cancer Patients: How Can Patients Be Assisted?. Front. Oncol..

[21] Gondal H., Abbas T., Choquette H., Le D., Chalchal H.I., Iqbal N., Ahmed S. (2022). Patient and Physician Satisfaction with Telemedicine in Cancer Care in Saskatchewan: A Cross-Sectional Study. Curr. Oncol..

[22] Alsabeeha N.H.M., Atieh M.A., Balakrishnan M.S. (2022). Older Adults’ Satisfaction with Telemedicine during the COVID-19 Pandemic: A Systematic Review. Telemed. J. e-Health.

[23] Pakanati V., Raol N., Ching Siong T., Govil N. (2022). Patient and physician satisfaction with telemedicine in pediatric otolaryngology. Int. J. Pediatr. Otorhinolaryngol..

[24] Kruse C.S., Krowski N., Rodriguez B., Tran L., Vela J., Brooks M. (2017). Telehealth and patient satisfaction: A systematic review and narrative analysis. BMJ Open.

[25] Rhoden P.A., Bonilha H., Harvey J. (2022). Patient Satisfaction of Telemedicine Remote Patient Monitoring: A Systematic Review. Telemed. J. e-Health.

